# Ho‘ouna Pono implementation: applying concept mapping to a culturally grounded substance use prevention curriculum in rural Hawai‘i schools

**DOI:** 10.1186/s43058-022-00359-2

**Published:** 2022-10-12

**Authors:** Kelsie H. Okamura, Scott K. Okamoto, Sarah Momilani Marshall, Steven Keone Chin, Pamela M. Garcia, Byron J. Powell, Kelly A. Stern, Sara J. Becker, David S. Mandell

**Affiliations:** 1 The Baker Center for Children and Families/Harvard Medical School, 53 Parker Hill Ave, Boston, MA 02120-3225 USA; 2 Department of Psychology, University of Hawai‘i at Mānoa, 2530 Dole Street, HI 96826 Honolulu, USA; 3 Population Sciences in the Pacific Program, University of Hawai‘i Cancer Center, 701 Ilalo Street, Honolulu, HI 96813 USA; 4 Department of Psychology, Hawai‘i Pacific University, 500 Ala Moana Blvd, Honolulu, HI 96813 USA; 5 Center for Mental Health Services Research, Brown School, Washington University in St. Louis, St. Louis, MO USA; 6 Center for Dissemination and Implementation, Institute for Public Health, Washington University in St. Louis, St. Louis, MO USA; 7 Division of Infectious Diseases, John T. Milliken Department of Medicine, School of Medicine, Washington University in St. Louis, St. Louis, MO USA; 8 Hawaiʻi State Department of Education, West Hawaiʻi School Based Behavioral Health Services, 74-5000 Puohulihuli St, Kailua-Kona, HI 96740 USA; 9 Center for Dissemination and Implementation Science, Northwestern University Feinberg School of Medicine, Chicago, USA; 10 Department of Psychiatry, University of Pennsylvania, 3535 Market Street, 3rd Fl, Philadelphia, PA 19104 USA

**Keywords:** Concept mapping, Rural schools, Culturally grounded prevention, Substance abuse

## Abstract

**Background:**

Despite their potential to ameliorate health disparities and address youth substance use, prevention programs have been poorly disseminated and implemented across Hawai‘i, which begs the question: *Why are effective prevention programs not being used in communities most in need of them?* Implementing and sustaining culturally grounded prevention programs is critical to address equitable healthcare and minimize health disparities in communities. The field of implementation science provides frameworks, theories, and methods to examine factors associated with community adoption of these programs.

**Method:**

Our project applies concept mapping methods to a culturally grounded youth drug prevention program with state level educational leadership in rural Hawai‘i schools. The goal is to integrate barrier and facilitator salience collected through teacher and school staff surveys and specific implementation strategies to regionally tailored implementation plans on Hawai‘i island. This protocol paper describes the concept mapping steps and how they will be applied in public and public-charter schools.

**Discussion:**

Improving prevention program implementation in rural schools can result in sustained support for populations that need it most. The project will integrate implementation science and culturally grounded methods in rural Hawai‘i, where most youth are of Native Hawaiian and Pacific Islander descent. This project addresses health disparities among Native Hawaiian and Pacific Islander youth and provides actionable plans for rural Hawai‘i communities to implement effective prevention programming.

Contributions to the literature

- This project uses culturally grounded and implementation science methods to address drug prevention in Native Hawaiian and Pacific Islander youth.
- Concept mapping methods will be used to engage educational leadership to sustain a drug prevention school-based curriculum in rural Hawaiʻi.
- Findings will extend implementation science methods to address populations that experience disparate health outcomes.

## Background

National and local epidemiological data consistently demonstrate the need for substance use prevention programs targeting Native Hawaiian and Pacific Islander (NHPI) youth. Few programs have been developed for these populations [1, 2], and those that have been developed rarely are disseminated and implemented in the communities where these youth live [3]. NHPI youth have significantly higher rates of drug use than their non-NHPI counterparts [4–6], and these differences are particularly pronounced among rural youth [7].

Some researchers have developed and evaluated drug prevention programs specifically for NHPI youth [1, 2]. The two most common approaches to developing culturally relevant programs are adaptation and grounded approaches [8]. Cultural adaptations involve modifying preventive content originally developed for different populations, while grounded approaches develop programs from the cultural values and beliefs specific to each culture. Studies examining the efficacy of culturally adapted prevention programs report mixed findings, prompting concerns that adaptation results in the loss of active program ingredients [9].

In contrast to culturally adapted approaches, culturally grounded approaches place the culture of the participant at the center of the preventive message. Content is designed based on the culture and social context of the targeted population [10]. Few culturally grounded prevention programs have been developed for and evaluated with NHPI youth [1, 11, 12]. Some research has suggested that prevention programs for NHPI youth should be developed using a grounded approach, given that drug prevention affects indigenous youth differently than other ethnocultural groups [13].

The Ho‘ouna Pono substance use prevention curriculum was developed using a culturally grounded approach with youth and community collaborators with a vested interest in promoting well-being on rural Hawai‘i Island [2, 11]. The Ho‘ouna Pono curriculum was born out of a request from the Hawai‘i State Prosecuting Attorney’s Office and in partnership with two local universities and the Hawai‘i Department of Education (HIDOE). This teacher-delivered, interactive curriculum aligns with Hawai‘i Content and Performance Standards for Health, grades six through eight. Intervention fidelity and implementation support is provided through an online virtual classroom and discussion board. The Ho‘ouna Pono curriculum has been developed, piloted, and evaluated across three consecutive NIDA-funded grants over the past 11 years in the State of Hawaiʻi (K01 DA019884, R34 DA031306, and R01 DA037836-01A1). More recent efforts have identified factors associated with Ho‘ouna Pono curriculum adoption, implementation, and sustainability.

Implementation science is the “scientific study of methods to promote the systematic uptake of research findings” [14]. Implementation science provides theories, frameworks, and methods to *study* and *implement* innovations in real-world settings. The Consolidated Framework for Implementation Research (CFIR [15]) is one such framework that emphasizes the dynamic and multi-contextual nature of implementation through multiple influences (e.g., settings, implementers, intervention characteristics). Understanding implementation through unified frameworks allows researchers to develop a common language around implementation barriers and facilitators. Applying implementation science methods to culturally grounded prevention allows communities and researchers to address all aspects of the *curriculum content* and *delivery* to ensure that programs make it into routine practice [14].

The current project uses concept mapping to inform regionally tailored implementation, adoption, and sustainability plans for the Ho‘ouna Pono curriculum. An ongoing project (R34 DA046735) will describe implementation barriers and facilitators, using survey and interview data collected through a previous efficacy study (R01 DA037836-01A1). Findings from a pilot study with 24 HIDOE members revealed both Ho‘ouna Pono strengths and barriers across all CFIR domains [16], including the inner setting (e.g., “There is a lack of HIDOE funding to support prevention curricula”), outer setting (e.g., “Marijuana use is socially acceptable on Hawai‘i island, diminishing the need”), and individual characteristics of the adopter (e.g., “Some HIDOE teachers are resistant to trying new curricula…because it feels like one more thing administrators want [us] to do in the classroom”).

Concept mapping is an implementation method that involves engaging implementers and the systematic mapping of salient opinions onto a framework for developing implementation strategies [17, 18]. It has been used to support youth behavioral health intervention implementation [19]. The present translational implementation study is timely given shifting priorities for diversity, equity, and inclusion across the research spectrum, and generalizable knowledge of implementation science within rural Hawai‘i youth prevention programs in school-based settings with an emphasis on NHPI youth populations. The results will help to further the prevention science field’s understanding of how to implement culturally grounded programs within school-based settings and allow for future studies on the impact of these plans on sustainability.

## Method

### Data available from parent trial

This mixed-methods study will analyze the salience of implementation barriers and facilitators building upon efforts of the parent grant (R34 DA046735). A set of 50 barriers and 27 facilitators were gathered through HIDOE member focus groups. Focus group members were asked open-ended questions about Ho‘ouna Pono implementation based on CFIR domains [16]. The current parent grant study asks participants to rate the 50 identified barriers on a five-point scale regarding the extent to which a particular barrier will *impact* implementation (1 = no impact, 2 = minimal impact, 3 = some impact, 4 = significant impact, and 5 = substantial impact) and the *difficulty* in overcoming these barriers (1 = very easy, 2 = easy, 3 = a little difficult, 4 = moderately difficult, and 5 = very difficult). Participants are asked to review the list of 27 facilitators and select their top three regarding the perceived effectiveness in addressing implementation. Surveys were administered to HIDOE teachers, staff, and administrators across schools on Hawai‘i Island at already occurring group meetings (e.g., faculty meetings). These schools have partnered with the project team through the development, pilot, and effectiveness studies. Informed consent and confidentiality have been carefully explained to all participants. This project has Institutional Review Board approval from the [University of Hawaiʻi at Mānoa, Hawaiʻi Pacific University, and HIDOE].

To date, we have collected 175 surveys from faculty, administrators, and support staff across 33 schools on Hawai‘i island. Preliminary examination of survey results indicate that the highest impact barriers relate to the inner setting (e.g., “There is lack of Hawaii DOE funding to support prevention curricula like Ho‘ouna Pono”) and outer setting (e.g., “Marijuana use is socially acceptable on Hawai‘i island, diminishing the need for Ho‘ouna Pono”) and the most difficult barriers related to inner setting and individual implementer characteristics (e.g., “Some Hawai‘i DOE teachers are resistant to trying new curricula like Ho‘ouna Pono, because it feels like ‘one more thing you want me to do in the classroom’”). The most commonly selected facilitators have centered mostly around intervention characteristics (e.g., “The curriculum is free,” “The curriculum is place-based, focused on the ‘local’ culture of Hawai‘i island”) and inner setting (e.g., “Ho‘ouna Pono is aligned with superintendent emphasis on student voice”).

### Concept mapping

We will use concept mapping to mathematically and visually understand relationships among the identified barriers and facilitators, with the goal of developing implementation strategies and regionally tailored plans. Concept mapping can help improve the selection and tailoring of implementation strategies [17, 18, 20] by providing a meaningful visual depiction of implementation concepts to participants [21–23]. This method allows organization of complex and diverse ideas into a clear and comprehensive framework to improve engagement [21].

**Fig. 1:**
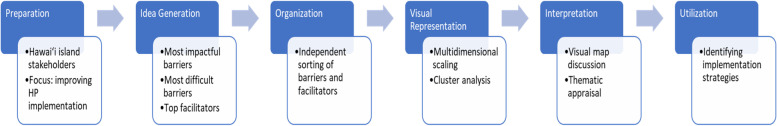
Concept mapping phases

Concept mapping of the Ho‘ouna Pono barriers and facilitators will include six stages (see Fig. 1): (1) preparation—selecting participants and defining conceptual focus, (2) generation of statements, (3) structuring of statements, (4) representation—visually and through multidimensional scaling and cluster analysis, (5) interpretation with participants, and (6) using the clusters for the development of implementation action plans. Implementation barriers and facilitators will be structured and represented visually (cf. [18]). Participants will be kept on task and group continuity encouraged via structured facilitation. We hypothesize that there will be several multi-contextual inner setting influences, with within school variations being more impactful than those at the individual teacher level.

In the first phase, HIDOE leadership at the complex (i.e., geographically grouped clusters of schools) and state level will participate in one-hour focus groups in which they discuss highly endorsed impactful and difficult barriers as well as frequently endorsed helpful facilitators. Survey data from the parent grant will be used to identify highly endorsed items. When possible, participants will be divided by HIDOE complex (e.g., Kaʻū-Kea‘au-Pāhoa) and may represent diverse roles (e.g., district education specialist, curriculum coordinator). Recruitment is based on previous Ho‘ouna Pono focus groups’ feedback indicating many implementation barriers at the inner and outer setting, which are typically out of individual teachers’ control. Participants will be chosen based on HIDOE leadership role to dually understand barrier and facilitator constructs from a unique perspective and increase engagement in implementation.

During the second phase, the barriers and facilitators identified through the parent grant will be presented to participants. Participants will review the items and generate additional barriers and facilitators based on their perspective. This step will be conducted through a preparation meeting or email communication given the regulations around in-person HIDOE research data collection. We will instruct participants to review barriers and facilitators and provide any feedback prior to the third grouping phase.

In the third phase, structuring of statements, participants will independently sort barriers and facilitators into concepts. This will be done through online groupwisdom software (Concept Systems Incorporated, 2013), and participants will be instructed to sort based on their understanding of Ho‘ouna Pono implementation. Each grouping must have more than one barrier or facilitator, and barriers and facilitators cannot be put into one large group or an additional miscellaneous group [22]. Participants also will rate each barrier and facilitator on its relative importance on a five-point scale.

In the fourth phase, representation, our team will use multidimensional scaling and cluster analysis to visually create common groupings based on similarity (i.e., how HIDOE leadership grouped items). Multidimensional scaling will be used with the sorting task to create a matrix of similarities between statements and stakeholder groupings. This will yield a two-dimensional solution that will serve as the input for a cluster analysis using Ward’s algorithm [24, 25] to define orthogonal clusters or groupings. Cluster analysis will adhere to the typical rule of approximately five statements per cluster and various solutions will be examined based on point map and interpretability of the clusters [21, 22]. Relative importance ratings will then be layered on to the cluster map to introduce average importance among participants into a cluster rating map.

The fifth phase, interpretation, will bring participants together to discuss the cluster rating and point maps to determine the label for each cluster. Participants will be shown the items within a cluster, asked to identify a label for the cluster, and the point map where items were physically located together. The final labeled cluster map will be presented to stakeholders with relative importance averages to solicit feedback on the extent to which the map visually represents their perceived contextual determinants and priorities. The cluster map will be compared against the factor structure emerging from the survey data analysis of HIDOE school faculty and staff.

In the sixth and final phase, the concept maps will be used to facilitate discussions on addressing the Ho‘ouna Pono implementation clusters with implementation strategies. Specifically, participants will brainstorm strategies to address Ho‘ouna Pono implementation and will also be guided to use the Expert Recommendations for Implementation Change (ERIC [20, 26, 27]) taxonomy as a resource to select implementation strategies. Cluster rating maps indicating relative importance will help to prioritize and sequence implementation strategies. This final phase will be the foundation for region-specific implementation plans to support the sustainability of the curriculum.

### Procedure

Recruitment for concept mapping focus groups will begin in Fall 2022 at the start of the HIDOE school year. This timing allows for the greatest representation of HIDOE leadership because many staff are 9- or 10-month employees. Participants will be selected based on their role and Ho‘ouna Pono involvement, through consultation with HIDOE leadership. Given COVID-19 health protocols and HIDOE policies on in-person data collection, these focus groups will be conducted online and will be guided by our previous school-based studies on Hawaiʻi island [28, 29]. Participants will be recruited through email invitation that will outline the rationale, importance of HIDOE leadership perspective, and time commitment. We expect that participants will engage in one asynchronous hour sorting and grouping barriers and facilitators, and two one-hour zoom meetings discussing cluster analysis findings. Participants will be compensated for each time point with a $50 gift card. Data integrity and security will be maintained through standardized procedures aligned with university standards with only trained staff and graduate students having data access. Data will be analyzed using Concept System Global Max for multidimensional scaling and cluster analysis, and SPSS for survey data analysis. When possible, focus groups will be audio recorded, transcribed verbatim, and archived for reference.

## Discussion

Implementation of evidence-based substance use prevention programs is particularly important for Native Hawaiian and Pacific Islander youth, who experience unique disparities related to substance use. The proposed project will address the need to understand HIDOE leadership perspectives on health and prevention curriculum implementation in rural Hawaiʻi. This study applies innovative implementation science methods such as concept mapping to develop multi-faceted implementation strategies. Concept mapping also will provide a richer understanding of how HIDOE leadership conceptualize implementation barriers and facilitators, which can inform future research examining school-based health standards and curricula.

Our findings will inform regionally tailored implementation plans based on empirically derived factors and clusters produced with concept mapping. In addition to the HIDOE leadership derived clusters emerging from concept mapping, the factor structure from teacher, counselor, and other school staff surveys will be examined using data from the parent study. Integrating these two classification types allows for a comprehensive approach to addressing and engaging school staff across the multiple contexts that intersect to support Ho‘ouna Pono. Moreover, the broader study examines two complementary methods to elucidating the impact of implementation barriers and facilitators. This will allow for pragmatic decisions in future research on feasibility of these methods to both engage and enhance implementation.

Of importance to the community, implementation strategy selection by HIDOE will be informed by region-specific barrier and facilitator findings. For example, it may be that within a complex, teachers and other school staff indicate that the most impactful barriers are related to school leadership turnover (e.g., principal transfers to another school). Meanwhile, HIDOE leadership cluster findings may suggest the most impactful barriers are teachers’ training and personal reluctance to engage in new curriculum given multiple demands. Implementation strategy selection will be guided by the Expert Recommendations for Implementing Change [26] and compiled into plans informed by implementation phases (e.g., Exploration, Preparation, Implementation, Sustainability [30]).

Several study limitations should be noted. First, generalizability is limited to the setting (i.e., rural Hawaiʻi island) and population (i.e., Native Hawaiian and Pacific Islander youth) so caution should be used when extending findings to urban populations within Hawaiʻi, the continental United States, and beyond. Second, we anticipate that COVID-19 safety procedures will continue to lessen; however, our team cannot anticipate the COVID-19 impact on HIDOE staff participation. Nevertheless, our study applies innovative implementation science methods to an under-researched health disparity population in the Pacific region. Barriers will be addressed through implementation strategies in regionally tailored action plans to enhance adoption and sustainability. These plans will be used to inform a hybrid type-II trial in a novel rural Hawaiʻi setting to elucidate effective implementation strategies of culturally grounded prevention interventions.

## Data Availability

Data sharing is not applicable to this article as no datasets were generated or analyzed during the current study. However, once completed, the datasets used and/or analyzed will be available from the corresponding author on reasonable request.

## Abbreviations

IAS: Implementation, adoption, and sustainability
NHPI: Native Hawaiian and Pacific Islander
CFIR: Consolidated Framework for Implementation Research
HIDOE: Hawai‘i Department of Education
